# Sex-related differences in the prognostic utility of inflammatory and thrombotic cardiovascular risk markers in patients with chest pain of suspected coronary origin

**DOI:** 10.1016/j.ijcha.2025.101600

**Published:** 2025-01-13

**Authors:** Dennis Winston T. Nilsen, Reidun Aarsetoey, Volker Poenitz, Thor Ueland, Pål Aukrust, Annika Elisabet Michelsen, Trygve Brugger-Andersen, Harry Staines, Heidi Grundt

**Affiliations:** a Stavanger University Hospital, Department of Cardiology, Stavanger, Norway; b University of Bergen, Department of Clinical Science, Bergen, Norway; c Thrombosis Research Center (TREC), Division of Internal Medicine, University Hospital of Northern Norway, Tromsø, Norway; d University of Oslo, Faculty of Medicine, Oslo, Norway; e Oslo University Hospital, Rikshospitalet, Research Institute of Internal Medicine, Oslo, Norway; f Oslo University Hospital, Rikshospitalet, Section of Clinical Immunology and Infectious Diseases, Oslo, Norway; g Stavanger Heart Center, Stavanger, Norway; h Sigma Statistical Services, Balmullo, United Kingdom of Great Britain and Northern Ireland; i Stavanger University Hospital, Department of Respiratory Medicine, Stavanger, Norway

**Keywords:** Gender/sex, Biomarkers, SERPINA3, D-dimer, acute coronary syndrome (ACS), cardiovascular disease (CVD) events/mortality

## Abstract

**Background:**

α1-antichymotrypsin (SERPINA3), high sensitivity C-reactive protein (hsCRP) and pentraxin 3 (PTX3) are acute phase proteins triggered by inflammation, whereas D-dimer, fibrin monomer and α2-antiplasmin are thrombo-fibrinolytic markers. Sex differences in relation to cardiovascular disease were investigated.

**Methods:**

A total of 871 consecutive patients (61.0 % males; females: 77.3 years, males 69.1 years) were included. Of these, 380 were diagnosed with an acute myocardial infarction (MI). Stepwise Cox regression models, applying normalized continuous log_e_/SD values, were fitted for the biomarkers with all-cause mortality, MI and stroke, respectively, and a composite endpoint within 7 years as the dependent variables.

**Results:**

Except for α2-antiplasmin, all biomarkers were significantly associated with all-cause mortality and the combined endpoint in the univariate analysis. None of the inflammatory biomarkers predicted all-cause mortality in females after multivariable adjustment but were significant predictors in males (SERPINA3: HR 1.34 (95 %CI 1.16–1.56), p < 0.0001. hsCRP: HR 1.19 (95 %CI 1.02–1.38), p = 0.027. PTX3: HR 1.22 [95 %CI 1.04–1.44], p = 0.018. The p-value for interaction suggests a sex difference in the prognostic weighting of SERPINA3 (p = 0.015). None of the thrombo-fibrinolytic biomarkers predicted all-cause mortality in males after adjustment, but D-dimer and fibrin monomer were significant predictors of all-cause mortality in females (HR 1.51 [1.29–1.78], p < 0.0001, and HR 1.28 [1.08–1.53] p = 0.005, respectively). A trend towards interaction for D-dimer (p = 0.07) may suggest a sex difference in its prognostic weighting.

**Conclusion:**

SERPINA3, hsCRP and PTX3 predicted long-term all-cause mortality in males but not in females. The opposite relationship was observed for D-dimer and fibrin monomer.

## 1 Introduction

The prevalence of cardiovascular disease (CVD) is lower but the rate of death and morbidity after MI is higher in women than in men [1], [2], [3], [4]. Several factors may be related to gender differences in clinical outcomes, such as hormones [5], [6], differences in physiological challenges and comorbidities [5], [6], [7], and sociocultural factors [8], as well as diagnostic and therapeutic factors [9].

Men may be more prone to arterial and venous thromboembolic disease than women [10], [11]. Natural estrogen in premenopausal women may exert protective effects against thromboembolism, whereas risk is increased by pregnancy and oral contraceptives. Postmenopausal women will no longer be protected and will carry a similar risk of thromboembolic disease as that of men, and notably, peroral estrogen, but not transdermal estrogen replacement therapy may increase their risk [10], [11], illustrating the complexity of the interaction between estrogen and thrombus formation. Moreover, the higher occurrence of non-obstructive coronary artery disease in women than in men, often associated with microvascular coronary dysfunction, further complicates the picture [5].

Based on these issues, sex differences in the prognostic utility of cardiovascular biomarkers may be of practical importance [12]. High sensitivity C-reactive protein (hsCRP) is generally regarded as a reliable marker of up-stream inflammation and has yielded valuable prognostic information in several studies [12], [13], [14]. α1-antichymotrypsin (SERPINA3) is a member of the serpin superfamily of proteinase inhibitors and inhibits a wide range of serine proteases, such as chymotrypsins, cathepsin G, and mast cell chymase [15], [16]. Like CRP, SERPINA3 is mainly produced in hepatocytes and behaves as an acute phase protein stimulated by proinflammatory cytokines, such as interleukin-6 (IL-6) [17]. Pentraxin 3 (PTX3) is a multimeric acute-phase protein and like CRP it belongs to the pentraxin superfamily [18], [19]. In contrast to CRP, PTX3 is produced mainly at the site of inflammation such as within an atherosclerotic lesion with neutrophils, macrophages, smooth muscle cells and endothelial cells as important cellular sources [19]. Although there are some data on CRP in relation to gender-related CVD outcomes [13], [14], reports on sex differences in the prognostic utility of SERPINA3 and PTX3 in relation to CVD are scarce.

In addition to inflammatory markers, molecules pertaining to ongoing thrombosis formation are also relevant in relation to gender differences in CVD prognosis. D-dimer and fibrin monomer may serve as valuable indicators of pro-thrombotic states [20], and the level of α2-antiplasmin (α2AP) may reflect ongoing inactivation of plasmin during breakdown of fibrin [21]. D-dimer was shown to serve as a predictor of long-term all-cause mortality in patients hospitalized for chest pain of suspected coronary origin [18]. In general, however, the use of these markers in relation to gender differences in CVD prognosis is incompletely understood.

To fill these knowledge gaps, we have investigated the prognostic utility of these inflammatory (i.e., hsCRP, SERPINA3 and PTX3) and thrombo-fibrinolytic (i.e., D-dimer, fibrin monomer and α2AP) biomarkers in relation to sex-related outcomes during 7 years follow-up in patients hospitalized for chest pain of suspected coronary origin.

## 2 Material and Methods

### 2.1 Study design and patient population

Risk Markers in the Acute Coronary Syndrome (RACS) (ClinicalTrials.gov Identifier: NCT00521976) included a total of 871 consecutive patients with chest pain of suspected coronary origin [18], [20]. All patients were admitted to Stavanger University Hospital, Norway, from November 2002 until September 2003. They were initially followed up prospectively for 2 years, then later for a further 5 years, increasing follow-up to a median of 7 years. Exclusion criteria were age < 18 years, prior inclusion, unwillingness or incapacity to provide informed consent. An acute myocardial infarction (AMI) was classified by troponin-T (TnT) measurements at baseline and 6 h after admission, employing a cut-off value > 50 ng/L and a typical rise and fall [16], [22].

### 2.2 Selected prognostic biomarkers

We investigated the sex-related prognostic utility of hsCRP, SERPINA3 and PTX3, D-dimer, fibrin monomer and α2-antiplasmin.

### 2.3 Collection of data and follow-up

Baseline data including information on demographics, smoking habits, clinical history, and general laboratory characteristics were collected at hospital admission. Clinical follow-up data were obtained prospectively from hospital- and public registries and by telephone interview at 30 days, 6, 12, 24 and 84 months, and, if needed, additional information was obtained from general practitioners and nursing homes [16], [18].

### 2.4 Endpoints

Separate endpoints at 7 years follow-up consisted of all-cause mortality, MI, and stroke, respectively. A combined endpoint consisting of all-cause mortality, MI or stroke was also analysed.

### 2.5 Ethics

Approval was obtained from the Regional Board of Research Ethics and by the Norwegian Health authorities. All patients gave their written informed consent. The study was conducted in accordance with the Helsinki declaration of 1971, as revised in 1983.

### 2.6 Blood sampling procedures and laboratory measurements

Blood was harvested by venepuncture immediately following hospital admission and a second blood sample was drawn 6–7 h later [18]. Measurements in serum of TnT, estimated glomerular filtration rate (eGFR), glucose and lipids were performed immediately after centrifugation at the laboratory for Medical Biochemistry at Stavanger University Hospital. Multiple aliquots of ethylene diamine tetraacetic acid (EDTA) plasma, citrated plasma and serum were frozen and stored at −80 °C for later duplicate measurements of prognostic biomarkers.

### 2.7 Troponin-T (TnT) measurements

In all patients, we applied a «rule-in rule-out» MI protocol using a modified “second generation” TnT assay for diagnosing an AMI [16], [22], [23]. The assay was based on a high-affinity cardiac-specific TnT isoform antibody (Roche Diagnostics, Germany), employing biotin-streptavidin coated microplates on a Elecsys TM Analyzer [22], [23]. The lower detection limit of the locally modified assay was 10 ng/L, with a cut-off level for MI of 50 ng/L and a coefficient of variation (CV) of 10 % [16], [22].

### 2.8 High-sensitivity C reactive protein (hsCRP) and pentraxin 3 (PTX3) measurements

An immunoturbidimetric assay was used for measurement of hsCRP in serum from 868 patients (Tina-quant® C-reactive protein (latex) high sensitive assay, Roche Diagnostics, Mannheim, Germany), performed on a Roche automated clinical chemistry analyzer (MODULAR P), with a detection limit of 0.03 mg/L. The between-assay CV was 3.45 % at 1.19 mg/L and 2.70 % at 0.43 mg/L [18]. PTX3 was analysed from 795 patients in EDTA plasma using a human PTX3 enzyme-linked immunosorbent assay (ELISA) kit (Perseus Proteomics Inc. Tokyo, Japan), performed on a BEP III analyser (Dade Behring, Germany). The PTX3 concentration was measured linearly between 0.1 and 20 ng/ml. The intra- and inter-assay CV were 2.9 % at 0.67 μg/L and 0.8 % at 15.91 μg/L, and 1.8 % at 0.66 μg/L and 4.1 % at 15.95 μg/L respectively [18].

### 2.9 Measurement of D-dimer and fibrin monomer

D-dimer was measured in citrated plasma from 820 patients by an immunoturbidimetric method on a coagulation analyzer with assay reagents from Biopool (Umea, Sweden). The reproducibility of the assay by intra-series CV was 2.2 %. The lowest detection limit was 100 μg/L [20]. Fibrin monomer was analyzed in citrated plasma from 810 patients by an ELISA system (ENZYMUN fibrin monomer, Roche Diagnostics, Mannheim, Germany). The minimum detection limit was 2.3 mg/L, and the within-run coefficients of variation were below 10.0 % [20].

### 2.10 Measurements of α1-antichymotrypsin (SERPINA3) and α2-antiplasmin (α2AP)

Plasma samples from 847 patients were analyzed for SERPINA3 (given in arbitrary units, AU) using antibodies from Abnova (Taipei, Taiwan) [16], and for α2AP (mg/L) using antibodies from R&D Systems (Minneapolis, MN, USA), applying a 384-well format with a combination of a SELMA (Jena, Germany) pipetting robot and a BioTek (Winooski, VT, USA) dispenser/washer. Absorption was read at 450 nm with wavelength correction set to 540 nm using an ELISA plate reader (BioTek). Intra- and inter-assay CV were < 10 %. EDTA blood was lacking in 65 patients and was replaced by citrated blood. Based on data from our own laboratory, measurements in EDTA and citrated plasma did not differ significantly.

#### 2.10.1 Statistical analysis

Descriptive statistics are presented as medians with interquartile range (25th – 75th percentile) for continuous data and as numbers and percentages for categorical data. Differences in baseline characteristics were assessed by the Kruskal-Wallis test for continuous data and the Chi-squared test for categorical data. Due to a skewed distribution, SERPINA3, hsCRP, PTX3, BNP and eGFR levels were logarithmically transformed to the base-e (log_e_) prior to analysis of continuous values and normalised by dividing by the standard deviation (SD).

Separate analyses were conducted by biomarker and clinical endpoint. The time to the first occurrence of the endpoint (for the composite endpoint the time to the first occurrence of MI, stroke or death) was recorded. Cox regression models were fitted for the clinical endpoints within the defined follow-up periods as the dependent variable. In the multivariable model the biomarker was forced into the model with baseline risk markers and baseline clinical risk factors added stepwise, including TnT  ≤ 10 ng/L rather than the AMI diagnostic level of TnT in the model (Table 1). The interaction between sexes was analyzed by fitting a multivariable Cox model as above but with sex and the biomarker sex interaction term forced into the model before adding confounders stepwise. The hazard ratios presented in the results section are 1-SD on the log scale.

**Table 1:** Baseline characteristics.

**Characteristics**	**Total n = 871**	**Female n = 340**	**Male n = 531**	**p-value**
***Biomarkers***				
**SERPINA3 (AU)**	**51.8 (43.4**–**62.4)**	**51.9 (43.7**–**63.5)**	**51.6 (43.1**–**61.4)**	**0.30**
hsCRP (mg/L)*	**4.0 (1.7**–**13.3)**	**3.9 (1.7**–**14.4)**	**4.0 (1.8**–**12.1)**	**0.97**
**PTX3 (μg/L)**	**5.9 (3.5**–**9.5)**	**6.3 (3.7**–**9.9)**	**5.7 (3.4**–**9.4)**	**0.070**
**D-dimer (mg/L)**	**191.0 (106.0**–**437.0)**	**234.0 (134.0**–**613.0)**	**170.0 (51.0**–**367.0)**	**<0.001**
**FM (mg/L)**	**3.1 (1.4**–**8.3)**	**3.6 (1.6**–**10.8)**	**2.7 (1.3**–**6.3)**	**0.002**
**α2AP (mg/L)**	**1.9 (1.2**–**2.4)**	**1.9 (1.2**–**2.5)**	**1.8 (1.1**–**2.4)**	**0.24**

***Baseline risk markers***				
Age (years)*	**72.6 (59.1**–**81.1)**	**77.3 (66.6**–**83.8)**	**69.1 (56.1**–**77.2)**	**<0.001**
BNP (ng/L)*	**97.0 (33.5**–**310.5)**	**117.0 (44.0**–**353.0)**	**79.0 (29.0**–**281.0)**	**0.003**
eGFR ml/min/1.73 m2*	**63.3 (48.8**–**75.7)**	**55.2 (42.9**–**66.3)**	**68.9 (55.9**–**80.8)**	**<0.001**
Tot. chol. (mmol/L)*	**5.2 (4.3**–**6.0)**	**5.5 (4.6**–**6.4)**	**5.0 (4.1**–**5.8)**	**<0.001**
**TnT release ≤ 10 ng/L**	**400 (45.9)**	**175 (51.5)**	**225 (42.4)**	**0.009**
**TnT release > 10 and ≤ 50 ng/L**	**91 (10.5)**	**43 (12.7)**	**48 (9.0)**	**0.090**
**TnT release > 50 ng/L (acute MI)**	**380 (43.6)**	**122 (35.9)**	**258 (48.6)**	**<0.001**
TnT release > 10 ng/L*	**471 (54.1)**	**165 (48.6)**	**306 (57.6)**	**0.009**

***Risk factors***				
Smoking*				**<0.001**
**Current smoker**	**229 (26.3)**	**60 (17.7)**	**169 (31.8)**	
**Ex smoker**	**315 (36.2)**	**72 (21.12)**	**243 (45.8)**	
**Never smoked**	**327 (37.5)**	**208 (61.2)**	**119 (22.4)**	
Hypertension*	**367 (42.1)**	**161 (47.4)**	**206 (38.78)**	**0.013**
Diabetes mellitus type I*	**9 (1.0)**	**4 (1.2)**	**5 (0.9)**	**0.74**
Diabetes mellitus type. II*	**112 (12.9)**	**45 (13.2)**	**67 (12.6)**	**0.79**

***History of heart disease***				
Angina pectoris*	**381 (43.7)**	**172 (50.6)**	**209 (39.4)**	**0.001**
Prior MI*	**290 (33.3)**	**103 (30.3)**	**187 (35.2)**	**0.13**
Previous CABG*	**88 (10.1)**	**20 (5.9)**	**68 (12.8)**	**<0.001**
Previous PCI*	**87 (10.0)**	**18 (5.3)**	**69 (13.0)**	**<0.001**
Heart failure*	**235 (27.0)**	**107 (31.5)**	**128 (24.1)**	**0.017**

**Treatment prior to admission**				
ACE inhibitors or ARB*	**295 (33.9)**	**130 (38.2)**	**165 (31.1)**	**0.029**
Beta-blockers*	**313 (35.9)**	**129 (37.9)**	**184 (34.7)**	**0.32**
Statins*	**298 (34.2)**	**102 (30.0)**	**196 (36.9)**	**0.036**
Acetylsalicylic acid (ASA)*	**328 (37.7)**	**133 (39.1)**	**195 (36.7)**	**0.47**

Table displaying baseline characteristics of the total population, and separately for females and males, respectively, with p-values for sex differences.

Abbreviations: SERPINA3 = α1-antichymotrypsin. hsCRP = high sensitivity C-reactive protein. PTX3 = pentraxin3.

FM = fibrin monomer. α2AP = α2-antiplasmin. BNP = Brain Natriuretic Peptide. eGFR = estimated glomerular filtration rate.

Tot chol = total cholesterol. MI = myocardial infarction. TnT = troponin-T. BMI = body mass index. ACE = angiotensin-converting enzyme.

ARB = angiotensin receptor blocker. *Potential confounders added in the models.

Statistics were performed using the statistical package SPSS version 25 (IBM Corp. Armonk, NY). All tests were 2-sided with a significance level of 5 % without multiplicity adjustment.

## 3 Results

### 3.1 Baseline characteristics

Females were considerably older than males (difference in median age of 8 years, p < 0.001). They had a significantly higher rate of hypertension and higher levels of total cholesterol and BNP and lower levels of eGFR, whereas men had a significantly higher BMI, higher rate of smoking, more often chronic ischemic heart disease (angina pectoris, previous percutaneous coronary intervention [PCI] and coronary artery bypass grafting [CABG]) (Table 1). Also, there was a significantly higher proportion of females than males with no TnT release (≤ 10 ng/L), and vice versa was observed in patients with acute MI at index admission (TnT > 50 ng/L). Whereas there was no difference between the sexes in SERPINA3, hsCRP, PTX3 and α2AP levels, D-dimer and fibrin monomer were significantly higher in females (Table 1).

### 3.2 Follow-up considerations

No patient was lost to follow-up in this observational study. Several potential confounders were considered, as highlighted by an asterix in the baseline table (Table 1). A hierarchy of confounders were noted and defined according to the step at which the confounder was entered in the multivariable Cox model. For both sexes combined, age was the first confounder fitted for all models, whereas eGFR did not enter any model. The second confounder depended on the endpoint. For all-cause mortality, BNP quartile was the second confounder with heart failure entered in the third step and TnT > 10 ng/L in the fourth.

In the following endpoint analysis of associations, biomarkers are grouped pathophysiologically into inflammatory biomarkers (Table 2) and thrombo-fibrinolytic biomarkers (Table 3), respectively. The tables show the association between each of the group-related biomarkers and 7 years (a) all-cause mortality and (b) composite endpoint consisting of all-cause mortality or MI or stroke. In the supplementary section, tables with results related to patients with an acute myocardial infarction at index hospitalization, have been added.

**Table 2:** Univariate and multivariable analysis of three inflammatory biomarkers, SERPINA3, hsCRP and PTX3, as predictors of 7 years all-cause mortality and the composite endpoint of all-cause mortality or MI or stroke, respectively, in females and males, respectively, admitted with chest pain of suspected coronary origin.

		SERPINA3	hsCRP	PTX3
		HR (95 % CI) p −value	HR (95 % CI) p-value	HR (95 % CI) p-value
**All-cause mortality**				
Female	Uni	1.21 (1.02–1.42) 0.026	1.22 (1.04–1.42) 0.014	1.35 (1.15–1.58) < 0.001
	Multi	0.99 (0.83–1.19) 0.92	0.98 (0.82–1.16) 0.80	1.05 (0.89–1.24) 0.57
Male	Uni	1.57 (1.35–1.82) < 0.001	1.51 (1.32–1.73) < 0.001	1.79 (1.56–2.06) < 0.001
	Multi	1.34 (1.16–1.56) < 0.001	1.19 (1.02–1.38) 0.027	1.22 (1.04–1.44) 0.018
Multi	Interaction	P = 0.015	P = 0.074	P = 0.14

**Composite EP**				
Female	Uni	1.22 (1.05–1.42) 0.010	1.20 (1.05 – 1.39) 0.010	1.24 (1.07–1.44) 0.004
	Multi	1.07 (0.91–1.26) 0.40	1.04 (0.89–1.22) 0.62	1.01 (0.86–1.18) 0.96
Male	Uni	1.35 (1.18–1.53) < 0.001	1.41 (1.25–1.60) < 0.001	1.60 (1.41–1.81) < 0.001
	Multi	1.17 (1.03–1.33) < 0.001	1.16 (1.01–1.33) 0.031	1.20 (1.04–1.39) 0.013
Multi	Interaction	P = 0.28	P = 0.17	P = 0.027

Uni = univariate analysis; Multi = multivariable analysis; SERPINA3 = α1-antichymotrypsin; hsCRP = high sensitivity C-reactive protein; PTX3 = Pentraxin3. EP = endpoint.

**Table 3:** Univariate and multivariable analysis of three thrombo-fibrinolytic biomarkers, D-dimer, fibrin monomer and α2-antiplasmin as predictors of 7 years all-cause mortality and the composite endpoint of all-cause mortality or MI or stroke, respectively, in females and males, respectively, admitted with chest pain of suspected coronary origin.

		D-dimer	Fibrin monomer	α2-AP
		HR (95 % CI) p-value	HR (95 % CI) p-value	HR (95 % CI) p-value
**All-cause mortality**				
Female	Uni	1.83 (1.60–2.10) < 0.001	1.75 (1.50–2.06) < 0.001	1.09 (0.90–1.32) 0.38
	Multi	1.51 (1.29–1.78) < 0.001	1.28 (1.08–1.53) 0.005	1.04 (0.85–1.29) 0.69
Male	Uni	1.47 (1.33–1.62) < 0.001	1.91 (1.65–2.21) < 0.001	0.99 (0.86–1.15) 0.94
	Multi	1.11 (0.95–1.30) 0.20	1.11 (0.94–1.31) 0.23	0.94 (0.81–1.10) 0.45
Multi	Interaction	P = 0.070	P = 0.57	P = 0.29

**Composite EP**				
Female	Uni	1.53 (1.34–1.74) < 0.001	1.42 (1.23–1.65) < 0.001	1.11 (0.93–1.31) 0.25
	Multi	1.20 (1.02–1.40) 0.027	1.12 (0.95–1.32) 0.18	1.12 (0.93–1.35) 0.23
Male	Uni	1.34 (1.22–1.48) < 0.001	1.56 (1.38–1.78) < 0.001	1.03 (0.90–1.17) 0.69
	Multi	0.98 (0.85–1.14) 0.78	0.94 (0.81–1.09) 0.40	0.92 (0.81–1.06) 0.24
Multi	Interaction	P = 0.21	P = 0.42	P = 0.17

Uni = univariate analysis; Multi = multivariable analysis; α2-AP = α2-antiplasmin. EP = endpoint.

### 3.3 Inflammatory biomarkers and sex-related outcomes at 7 years follow-up

#### 3.3.1 Analysis of all-cause mortality and composite endpoint

In the univariate analysis, SERPINA3, hsCRP and PTX3 were significantly associated with 7 years all-cause mortality and the composite endpoint in both sexes, but remained significant for both endpoints only in males after multivariable adjustment (Table 2).

For all-cause mortality, the p-values for interaction in the multivariable analysis would suggest a significant sex difference in the prognostic weighting of SERPINA3 (p = 0.015), with a trend for hsCRP (p = 0.074), whereas no significant difference in interaction was noted for PTX3 (p = 0.14). (Table 2).

For the composite endpoint, the p-value for interaction in the multivariable analysis demonstrates a significant sex difference in the prognostic weighting of PTX3 (p = 0.027), suggesting that PTX3 acts independently as a predictor in males with respect to total death or MI or stroke (Table 2).

#### 3.3.2 Analysis of MI and stroke

HsCRP and PTX3 in males were significantly associated with MI in the unadjusted analysis, whereas only hsCRP remained significant after adjustment, but the p-value for interaction related to sex was non-significant (Supplementary Table 1). None of the biomarkers were associated with stroke (Supplementary Table 1).

### 3.4 Thrombo-fibrinolytic biomarkers and sex-related outcome at 7 years follow-up

#### 3.4.1 Analysis of all-cause mortality and composite endpoint

In the univariate analysis, D-dimer and fibrin monomer were each found to be significantly associated with 7 years all-cause mortality and the composite endpoint, respectively, in both sexes (Table 3). After multivariable adjustment, these associations remained significant for all-cause mortality only in females, among whom D-dimer was also shown to be significantly associated with the combined endpoint (Table 3). In contrast, α2AP was not found to be associated with these endpoints (Table 3).

For all-cause mortality, the p-value for interaction in the multivariable analysis was recorded as significant for D-dimer at 5 years follow-up (p = 0.01), with only a borderline trend at 7 years follow-up (p = 0.07), however, still showing a persistently higher HR in females as compared to males, whereas no difference in interaction was noted for fibrin monomer (p = 0.57) and α2-antiplasmin (p = 0.29) (Table 3). For the combined endpoint, all p-values for interaction in the multivariable analysis were non-significant (Table 3).

#### 3.4.2 Analysis of MI and stroke

In the univariate analysis, D-dimer and fibrin monomer in males were significantly associated with MI, but no association was obtained for any of the biomarkers including α2-antiplasmin, after adjustment, irrespective of sex (Supplementary Table 2). None of the biomarkers were associated with stroke (Supplementary Table 2).

### 3.5 All-cause mortality and composite endpoint in acute MI patients at 7 years follow-up

In patients with an acute MI (TnT release > 50 ng/L) at baseline, unadjusted SERPINA3 was significantly associated with all-cause mortality only in males at 7 years follow-up, but the association was weakened and borderline significant (p = 0.051) in the multivariable analysis, and presented with a non-significant p-value for interaction between the biomarker and sex (p = 0.17) (Supplementary Table 3).

The multivariable association between SERPINA3 and all-cause mortality was even stronger in male patients with no MI at index admission [HR 1.61 (95 %CI 1.30–1.99), p < 0.001, with a p-value of 0.048 for interaction between sexes].

Although significant in favor of males in the univariate analysis, multivariable adjustment showed no association between SERPINA3 and the combined endpoint. In the univariate analysis, hsCRP and PTX3 in males but not in females were associated with both endpoints, but no longer so after adjustment.

Unadjusted D-dimer was associated with all-cause mortality and the composite endpoint in both sexes, but acted as an independent predictor only in females, with a p-value of 0.007 and 0.035, respectively. However, the HR for these biomarkers did not differ significantly between sexes, thus no interaction was noted (Supplementary Table 4). In the univariate analysis, fibrin monomer was associated with both endpoints in both sexes, but had no independent prognostic value in AMI patients, irrespective of sex, and α2-antiplasmin failed entirely to predict outcome (Supplementary Table 4).

### 3.6 Confounders included in models pertaining to sex-related biomarkers

The order that clinical confounders were added to the models for all-cause mortality by sex and by the sex-related biomarkers, SERPINA3 and D-dimer, are shown in Supplementary Table 5. Similarly, confounders included in the models for the composite endpoint by sex and PTX3 are presented in Supplementary Table 6.

## 4 Discussion

In the current study we have evaluated the prognostic utility of inflammatory and thrombo-fibrinolytic biomarkers in relation to gender. There were several sex differences. At admission, females were considerably older than males and had higher BNP levels, whereas males exhibited a higher rate of previous ischemic heart disease. There was no baseline difference in SERPINA3, hsCRP, PTX3 and α2-antiplasmin, respectively, whereas D-dimer and fibrin monomer, respectively, were significantly higher in women as compared to men, and for D-dimer, a similar pattern has previously been reported [24], [25], [26], [27], [28].

In the adjusted analysis, all the selected inflammatory biomarkers in males were significantly associated with all-cause mortality and a composite endpoint consisting of all-cause mortality or MI or stroke at 7 years follow-up. An “opposite pattern” for D-dimer and fibrin monomer was seen in females for all-cause mortality and the former also predicted the composite endpoint, whereas α2-AP was of no predictive value.

Observation of a sex difference in prognostic weighting indicates that SERPINA3 is a sex-related long-term predictor of all-cause mortality in men and that D-dimer similarly predicts long-term all-cause mortality in women hospitalized with chest pain of suspected coronary origin. Only SERPINA3 was found to predict all-cause mortality limited to the male population with an acute MI at index admission, reaching borderline significance (multivariable model, p = 0.051) despite the presence of an acute phase reaction which is bound to mask its utility as a prognostic marker.

Also, limited to the male population of the entire group of patients, only hsCRP was found to independently predict future MI, but no sex preference in prognostic weighting was noted, as judged by the p-value for interaction.

During recent years, hsCRP is recognized as a global inflammatory biomarker of choice for prediction of future cardiovascular events in men [13] and women [14]. In our study of patients with suspected acute coronary heart disease, the inflammatory potential at admission was similar in both sexes, although females were 8 years older than males, whereas other studies based on presumptively healthy populations report a higher level of hsCRP in women as compared to men [29], [30]. This discrepancy is most likely due to the presence of cardiovascular disease among our patients and a lower proportion of females as compared to males presenting with an acute MI.

Our study results indicate that both SERPINA3 and hsCRP also may serve as long-term predictors of a composite endpoint consisting of all-cause mortality or MI or stroke in men admitted to hospital with chest pain of suspected coronary origin. Both biomarkers are acute phase proteins produced by hepatocytes, mainly mediated by the inflammatory cytokine IL-6 [17] and at least for SERPINA3 also IL-1 [31]. Its production is not restricted to hepatocytes, but also involves the brain, skin and genital organs [31]. In addition, SERPINA3 is more directly involved in extracellular matrix remodeling than CRP [31], [32]. If differences in regulation, production and function can explain the predominant prognostic utility of SERPINA3 as compared to CRP in men is, however, not clear.

PTX3 has previously been shown to provide valuable prognostic information related to long-term all-cause mortality in our studied patient group [18]. It surpassed hsCRP as a short-term predictor of mortality 3 months after an MI with ST-elevation [19]. In our study, PTX3 had a greater prognostic ability for males than for females. In fact, PTX3 surpasses SERPINA3 and hsCRP, in that order, as a long-term prognostic marker of the endpoint consisting of all-cause mortality or MI or stroke in men. Although CRP and PTX3 belong to the pentraxin family, PTX3 is, in contrast to CRP, produced at the site of vascular inflammation [33], potentially promoting its strength as a prognostic biomarker in certain inflammatory disorders like CAD and its complications [18]. Notwithstanding, the sex-related prognostic utility in men of SERPINA3 and PTX3 in a population admitted with suspected coronary chest pain has to the best of our knowledge not previously been reported and will require verification in future studies.

We have previously shown that D-dimer serves as a good predictor of long-term mortality [18], confirmed in the much larger LIPID study with 16 years follow-up [34] and also shown earlier in a large 4.2 year follow-up study of presumably healthy individuals [35]. In our present analysis, D-dimer, and to some degree also fibrin monomer, was found to be a sex-specific predictor of all-cause mortality in women admitted with suspected coronary chest pain, in line with other sex-specific risk factors of atherothrombotic disease in females [10], [11]. As compared to men, D-dimer in females appears to be a better predictor of risk of venous thromboembolism recurrence [27]. Reports specifically linking D-dimer to increased risk of CVD in women as compared to men are scarce. A cross-sectional correlation was noted between D-dimer and coronary event rates in women in the MONICA Optional Haemostasis Study [24]. In the prospective, observational Cardiovascular Health Study cohort study of risk factors in community-dwelling adults aged 65 years and older, D-dimer was identified as a risk factor of future CVD during 9 years follow-up, but did not appear to be a sex-specific risk factor [36]. These population studies, however, recruited healthy elderly subjects that differ from our population which included patients with chest pain of suspected coronary origin. Whether D-dimer and potentially also fibrin monomer could represent female specific risk markers in subjects presenting with coronary-related chest pain, however, needs to be verified in forthcoming studies.

### 4.1 Strengths and limitations

The RACS registry was established in 2002 with on-site invasive treatment available when required and with well-established prophylactic treatment with aspirin and statins in CHD patients. No patient was lost to follow-up. Furthermore, we present a direct comparison of several relevant biomarkers. AMI patients were sorted using a robust modified second generation TnT assay. The use of medication was recorded only at admission. Sampling of the biomarkers was limited to one draw, which was performed immediately following hospital admission, in order to be ahead of a major rise in acute phase reactants. An important limitation of the present study is that 56.4 % of the patients had no definitive MI diagnosis, at least partly reflecting a less sensitive TnT assay than used today. Thus, no definite diagnosis related to the origin of chest pain was pursued in patients with non-AMI, which will limit the relevance of our findings in relation to AMI management. However, the medication in non-AMI patients reflects a high frequency of patients with established CHD in this subpopulation [16]. Interestingly, SERPINA3 exerted a highly significant prognostic impact in the non-AMI male population, which is relevant to patients with acute chest pain of suspected coronary origin and no definite AMI, even in the era of high-sensitivity assays. Furthermore, all subjects were recruited from a Norwegian population. Therefore, our results may not necessarily be generalizable. The lack of recorded cardiac mortality at seven years follow-up limits the conclusion related to combined long-term cardiovascular events. Body Mass Index (BMI) was not reported during the initial phase of the study and not included in the multivariable model. Finally, the median age of the females was high, indicating that most of these were post-menopausal. Future studies should also include a larger number of premenopausal women.

## 5 Conclusions

In the present patient population presenting at the emergency department with chest pain of suspected coronary origin, SERPINA3 was found to be a robust sex-specific long-term predictor of all-cause mortality in men. Its utility as a prognostic marker of all-cause mortality may surpass that of hsCRP, when measured in male patients with acute chest pain of suspected coronary origin. In contrast, D-dimer appears to be a long-term predictor of all-cause mortality in women and is already frequently measured in admitted chest pain patients.

There is an urgent need for gender specific biomarkers in acute coronary chest pain patients, and further and larger studies should be performed to verify if SERPINA3 and D-dimer satisfy the criteria as such.

## Sources of Funding

This work was supported by the Western Norway Regional Health Authority and Stavanger University Hospital, Norway.

## ClinicalTrials.gov Identifier

NCT00521976

## CRediT authorship contribution statement

**Dennis Winston T. Nilsen:** Writing – original draft, Visualization, Validation, Supervision, Resources, Project administration, Methodology, Investigation, Funding acquisition, Conceptualization. **Reidun Aarsetoey:** Writing – review & editing, Validation, Methodology, Investigation. **Volker Poenitz:** Writing – review & editing, Validation, Methodology, Investigation. **Thor Ueland:** Writing – review & editing, Visualization, Supervision, Methodology, Investigation. **Pål Aukrust:** Writing – review & editing, Validation, Supervision, Methodology, Investigation. **Annika Elisabet Michelsen:** Writing – review & editing, Validation, Methodology, Investigation. **Trygve Brugger-Andersen:** Writing – review & editing, Validation, Methodology, Investigation. **Harry Staines:** Writing – review & editing, Validation, Supervision, Methodology, Investigation, Formal analysis, Data curation. **Heidi Grundt:** Writing – review & editing, Validation, Supervision, Methodology, Investigation, Data curation.

## Declaration of competing interest

The authors declare that they have no known competing financial interests or personal relationships that could have appeared to influence the work reported in this paper.
