# Supplementary material for: Sex-related differences in the prognostic utility of inflammatory and thrombotic cardiovascular risk markers in patients with chest pain of suspected coronary origin
Source: Int J Cardiol Heart Vasc. 2025 Jan 13;56:101600. doi: 10.1016/j.ijcha.2025.101600 (PMC11782882; doi:10.1016/j.ijcha.2025.101600)
Supplement: Supplementary Data 1 [file mmc1.docx]

**Supplementary Table 1.** Univariate and multivariable analysis of three inflammatory biomarkers, SERPINA3, hsCRP and PTX3, as predictors of 7 years MI and stroke, respectively, in females and males, respectively, admitted with acute chest pain of suspected coronary origin.

|  |  | SERPINA3 | hsCRP | PTX3 |
| --- | --- | --- | --- | --- |
|  |  | HR (95% CI) p-value | HR (95% CI) p-value | HR (95% CI) p-value |
| **MI** | |  |  |  |
| Female | Uni | 1.24 (0.99-1.56) 0.066 | 1.21 (0.98-1.50) 0.077 | 1.10 (0.87-1.38) 0.44 |
|  | Multi | 1.06 (0.84-1.36) 0.61 | 1.03 (0.81-1.31) 0.82 | 0.83 (0.66-1.06) 0.13 |
| Male | Uni | 1.19 (1.00-1.43) 0.054 | 1.43 (1.21-1.69) <0.001 | 1.47 (1.23-1.75) <0.001 |
|  | Multi | 0.98 (0.81-1.18) 0.81 | 1.26 (1.06-1.51) 0.010 | 1.11 (0.91-1.36) 0.31 |
| Multi | Interaction | P=0.80 | P=0.24 | P=0.12 |
| **Stroke** | |  |  |  |
| Female | Uni | 1.13 (0.77-1.67) 0.52 | 0.93 (0.63-1.37) 0.72 | 0.97 (0.64-1.45) 0.87 |
|  | Multi | 1.06 (0.69-1.61) 0.80 | 0.82 (0.54-1.26) 0.37 | 0.75 (0.47-1.20) 0.23 |
| Male | Uni | 1.30 (0.87-1.95) 0.20 | 1.10 (0.75-1.61) 0.63 | 1.25 (0.83-1.88) 0.29 |
|  | Multi | 1.22 (0.82-1.82) 0.33 | 1.01 (0.66-1.53) 0.98 | 0.96 (0.61-1.51) 0.86 |
| Multi | Interaction | P=0.51 | P=0.46 | P=0.54 |

Uni=univariate analysis; Multi=multivariable analysis; SERPINA3= α1-antichymotrypsin; hsCRP=high sensitivity C-reactive protein; PTX3=Pentraxin3. MI=myocardial infarction.

**Supplementary Table 2.** Univariate and multivariable analysis of three thrombo-fibrinolytic biomarkers, D-dimer, fibrin monomer and α2-antiplasmin as predictors of 7 years MI and stroke, respectively, in females and males, respectively, admitted with chest pain of suspected coronary origin.

|  |  | D-dimer | Fibrin monomer | α2-AP |
| --- | --- | --- | --- | --- |
|  |  | HR (95% CI) p-value | HR (95% CI) p-value | HR (95% CI) p-value |
| **MI** | |  |  |  |
| Female | Uni | 1.17 (0.93-1.48) 0.18 | 1.09 (0.86-1.38) 0.47 | 1.32 (1.00-1.75) 0.054 |
|  | Multi | 0.78 (0.57-1.06) 0.12 | 0.87 (0.66-1.13) 0.28 | 1.26 (0.94-1.70) 0.12 |
| Male | Uni | 1.29 (1.12-1.48) <0.001 | 1.45 (1.21-1.72) <0.001 | 1.08 (0.90-1.31) 0.41 |
|  | Multi | 0.87 (0.70-1.08) 0.21 | 0.97 (0.79-1.18) 0.75 | 0.97 (0.80-1.17) 0.75 |
| Multi | Interaction | P=0.63 | P=0.65 | P=0.19 |
| **Stroke** | |  |  |  |
| Female | Uni | 1.46 (0.98-2.16) 0.062 | 1.15 (0.77-1.73) 0.50 | 1.31 (0.82-2.11) 0.26 |
|  | Multi | 1.08 (0.68-1.73) 0.75 | 0.81 (0.53-1.22) 0.31 | 1.79 (0.98-3.26) 0.057 |
| Male | Uni | 0.99 (0.65-1.50) 0.96 | 1.07 (0.69-1.66) 0.75  0.74 (0.47-1.17) 0.20 | 1.05 (0.70-1.59) 0.81 |
|  | Multi | 0.70 (0.41-1.20) 0.20 | 0.74 (0.47-1.17) 0.20 | 1.04 (0.68 1-59) 0.85 |
| Multi | Interaction | P=0.26 | P=065 | P=0.24 |

Uni=univariate analysis; Multi=multivariable analysis; α2-AP=α2-antiplasmin. MI=myocardial infarction

**Supplementary Table 3.** Univariate and multivariable analysis of three inflammatory biomarkers, SERPINA3, hsCRP and PTX3, as predictors of 7 years all-cause mortality and the composite endpoint of all-cause mortality or MI or stroke, respectively, in females and males, respectively, admitted with an acute myocardial infarction at index admission.

|  |  | SERPINA3 | hsCRP | PTX3 |
| --- | --- | --- | --- | --- |
|  |  | HR (95% CI) p-value | HR (95% CI) p-value | HR (95% CI) p-value |
| **All-cause mortality** | |  |  |  |
| Female | Uni | 1.11 (0.85-1.46) 0.44 | 1.16 (0.90-1.49) 0.27 | 1.07 (0.81-1.42) 0.65 |
|  | Multi | 0.96 (0.72-1.29) 0.81 | 1.00 (0.75-1.33) 0.97 | 0.90 (0.67-1.22) 0.50 |
| Male | Uni | 1.43 (1.16-1.76) 0.001 | 1.53 (1.25-1.86) <0.001 | 1.84 (1.52-2.24) <0.001 |
|  | Multi | 1.24 (1.00-1.53) 0.051 | 1.14 (0.91-1.42) 0.24 | 1.19 (0.94-1.49) 0.15 |
| Multi | Interaction | P=0.17 | P=0.36 | P=0.15 |
| **Composite EP** | |  |  |  |
| Female | Uni | 1.17 (0.92-1.48) 0.21 | 1.16 (0.93-1.45) 0.18 | 1.01 (0.79-1.29) 0.95 |
|  | Multi | 1.09 (0.86-1.39) 0.49 | 1.02 (0.79-1.30) 0.90 | 0.92 (0.71-1.20) 0.55 |
| Male | Uni | 1.23 (1.03-1.46) 0.024 | 1.45 (1.22-1.73) <0.001 | 1.60 (1.34-1.91) <0.001 |
|  | Multi | 1.07 (0.90-1.28) 0.45 | 1.13 (0.92-1.39) 0.79 | 1.18 (0.97-1.44) 0.11 |
| Multi | Interaction | P=0.95 | P=0.35 | 0.09 |

Uni=univariate analysis; Multi=multivariable analysis; SERPINA3= α1-antichymotrypsin; hsCRP=high sensitivity C-reactive protein; PTX3=Pentraxin3. EP=endpoint.

**Supplementary Table 4.** Univariate and multivariable analysis of three thrombo-fibrinolytic biomarkers, D-dimer, fibrin monomer and α2-antiplasmin as predictors of 7 years all-cause mortality and the composite endpoint of all-cause mortality or MI or stroke, respectively, in females and males, respectively, admitted with an acute myocardial infarction at index admission.

|  |  | D-dimer | Fibrin monomer | α2-AP |
| --- | --- | --- | --- | --- |
|  |  | HR (95% CI) p-value | HR (95% CI) p-value | HR (95% CI) p-value |
| **All-cause mortality** | |  |  |  |
| Female | Uni | 1.79 (1.42-2.27) <0.001 | 1.80 (1.37-2.35) <0001 | 1.04 (0.77-1.40) 0.81 |
|  | Multi | 1.43 (1.10-1.85) 0.007 | 1.31 (0.95-1.82) 0.10 | 1.02 (0.75-1.40) 0.89 |
| Male | Uni | 1.39 (1.20-1.61) <0.001 | 1.93 (1.56-2.39) <0.001 | 0.82 (0.67-1.00) 0.055 |
|  | Multi | 1.02 (0.81-1.28) 0.89 | 1.06 (0.82-1.35) 0.67 | 0.84 (0.69-1-1.03) 0.09 |
| Multi | Interaction | P=0.087 | P=0.31 | P=0.30 |
| **Composite EP** | |  |  |  |
| Female | Uni | 1.42 (1.14-1.78) 0.002 | 1.40 (1.10-1.78) 0.007 | 1.10 (0.84-1.43) 0.50 |
|  | Multi | 1.30 (1.02-1.66) 0.035 | 1.24 (0.96-1.61) 0.11 | 1.14 (0.87-1.51) 0.35 |
| Male | Uni | 1.23 (1.07-1.42) 0.004 | 1.46 (1.22-1.75) <0.001 | 0.94 (0.78-1.14) 0.56 |
|  | Multi | 0.91 (0.74-1.139 0.40 | 0.90 (0.73-1.10) 0.30 | 0.97 (0.79-1.18) 0.75 |
| Multi | Interaction | P=0.14 | P=0.23 | P=0.42 |

Uni=univariate analysis; Multi=multivariable analysis;; α2-AP=α2-antiplasmin. EP=endpoint.

**Supplementary Table 5.** Stepwise presentation of confounders included in the models for all-cause mortality by sex and the sex-related biomarkers, SERPINA3 and D-dimer, arranged by order of entering the Cox regression model.

|  |  | |  | **All-cause mortality** | |  |  | | |
| --- | --- | --- | --- | --- | --- | --- | --- | --- | --- |
|  | **Males** | |  | **SERPINA 3** | |  | **Females** | | |
| **Step** | **Confounder** | **Score Chi-Square** | **Degrees of freedom** | **P-value** | **Confounder** | **Score Chi-Square** | | **Degrees of freedom** | **P-value** |
| **1** | Age | 142.97 | 1 | < 0.0001 | Age | 102.59 | | 1 | < 0.0001 |
| **2** | BNP quartile | 39.67 | 3 | < 0.0001 | TnT > 10 ng/L | 7.78 | | 1 | 0.005 |
| **3** |  |  |  |  | Heart failure | 8.16 | | 1 | 0.004 |
| **4** |  |  |  |  | Beta blockers | 4.44 | | 1 | 0.035 |

|  |  | |  | **D-dimer** | |  |  | | |
| --- | --- | --- | --- | --- | --- | --- | --- | --- | --- |
| **Step** | **Confounder** | **Score Chi-Square** | **Degrees of freedom** | **P-value** | **Confounder** | **Score Chi-Square** | | **Degrees of freedom** | **P-value** |
| **1** | BNP quartile | 138.77 | 3 | < 0.0001 | Age | 72.54 | | 1 | < 0.0001 |
| **2** | Age | 57.46 | 1 | < 0.0001 | Heart failure | 12.25 | | 1 | 0.0005 |
| **3** | Heart failure | 4.19 | 1 | 0.041 | Beta-blockers | 9.55 | | 1 | 0.002 |

BNP=Brain Natriuretic Peptide.

**Supplementary Table 6.** Stepwise presentation of confounders included in the models for the composite endpoint by sex and the sex-related biomarker PTX3 for males as compared to females, arranged by order of entering the Cox regression model.

|  |  | |  | **Composite endpoint**  **(Death or MI or stroke)** | |  |  | | |
| --- | --- | --- | --- | --- | --- | --- | --- | --- | --- |
|  | **Males** | |  | **Pentraxin 3 (PTX3)** | |  | **Females** | | |
| **Step** | **Confounder** | **Score Chi-Square** | **Degrees of freedom** | **P-value** | **Confounder** | **Score Chi-Square** | | **Degrees of freedom** | **P-value** |
| **1** | Age | 99.26 | 1 | < 0.0001 | Age | 76.72 | | 1 | < 0.0001 |
| **2** | BNP-quartile | 19.79 | 3 | < 0.0001 | TNT > 10 ng/L | 16.42 | | 1 | < 0.0001 |
| **3** | CABG | 6.84 | 1 | 0.009 | DM2 | 14.06 | | 1 | 0.0002 |
| **4** | TnT > 10 ng/L | 5.65 | 1 | 0.017 | Angina pectoris | 6.30 | | 1 | 0.012 |
| **5** |  |  |  |  | ACEI/ARB | 5.89 | | 1 | 0.015 |

DM2=Diabetes mellitus type 2. BNP=Brain Natriuretic Peptide. TnT= Troponin T.

CABG=Coronary Artery Bypass Grafting. ACEI=Angiotensin Converting Enzyme Inhibitor. ARB=Angiotensin Receptor Blocker.
